# Tingling, Numbness, and Underlying Malignancy: A Case Report of Glioblastoma Multiforme

**DOI:** 10.7759/cureus.76081

**Published:** 2024-12-20

**Authors:** Ritika Bhatia, Himanshi Singh, Jafor Sadeque, Kushagra Mathur

**Affiliations:** 1 General Medicine, Dartford and Gravesham NHS Trust, Dartford, GBR; 2 General Medicine, Chelsea and Westminster NHS Foundation Trust, London, GBR

**Keywords:** diagnostic dilenna, glioblastoma tumour, intracerebral mass, intracerebral metastasis, neurology and neuro-oncology

## Abstract

Glioblastoma multiforme (GBM) is a World Health Organisation (WHO) grade IV glioma originating from astrocytes. It is the most common malignant primary tumour of the brain and central nervous system (CNS) and is associated with fast progression and violent local spread, with a median overall survival of approximately 15 months after diagnosis. Due to its late and varied presentation, it is often diagnosed only after it has grown considerably. The symptomatology can vary from the individual being completely asymptomatic to mild sensory or sensorimotor symptoms. The symptoms usually arise due to compression of the fibres rather than pressure on vital structures.

We discuss a case of a 72-year-old male who presented with complaints of tingling in the left upper limb for one day, improving spontaneously. A noncontrast CT head was nondiagnostic, and an MRI reported a likely metastatic lesion, and further imaging was advised. A detailed history was taken from an oncology point of view but the patient denied any weight loss, fever, recent travel, and family history of cancers. The scans were discussed at a neuroradiology multidisciplinary team (MDT) discussion an MRI with contrast was planned. After a thorough discussion and further review by neuroradiology consultants, the patient was diagnosed with a grade IV glioblastoma - a primary tumour of the brain - which was confirmed by a biopsy and immunohistochemistry. He was treated with two cycles of radiotherapy initially, followed by chemotherapy. Unfortunately, he died eight months after the start of the treatment due to a massive pulmonary embolism, complicated by nosocomial infection.

This report highlights the importance of early diagnosis and treatment for patients with glioma. It also sheds light on the symptomatology and difficulties faced in the diagnosis of gliomas. Treating physicians should adopt an MDT approach in such cases and discuss the various possibilities and differentials.

## Introduction

Glioblastoma multiforme (GBM) is an aggressive and devastating malignancy originating from astrocytic glial cells. It is the most common malignant primary tumour of the brain and central nervous system (CNS), accounting for 48.6% of all brain tumours [1,2]. It is characterised by fast progression and an unfavourable prognosis, with a median overall survival of approximately 15 months after diagnosis [1-3]. GBM occurs more frequently in men, with 5.87 cases per 100,000 cases [4]. Most GBM cases are located in the frontal, temporal, and parietal lobes and may present as multiple lesions. Nearly half of these cases were diagnosed in patients older than 65 years [5], peaking between the ages of 75 and 84 (as reported in the Central Brain Tumor Registry of the United States (CBTRUS) in 2013, 2017, and 2020) before declining after 85 years [6]. 

The clinical presentation of GBM is diverse, with frequent symptoms such as intracranial hypertension (30%), motor impairments (20%), seizures (20%), altered consciousness (17%), confusion (15%), vision problems (13%), and speech difficulties (13%) [7]. Atypical presentations have been reported, such as lack of hand coordination and dysarthria [8]. GBM commonly presents atypically.

Several reports have described individuals presenting with uncommon symptoms, thus making it difficult for physicians to diagnose. Zhang et al. have described a patient who was inappropriately treated with antibiotics for intracerebral infections due to the MRI findings before being diagnosed with GBM after a biopsy [9]. Their patient received a similar treatment to ours. Sanli et al. have discussed three different cases that presented with unusual symptoms due to underlying GBM. One patient had left arm numbness but was misdiagnosed and treated for ulnar nerve entrapment, and later found to have advanced GBM when the symptoms did not improve. Another case report by Ekezie et al. involved a 15-year-old male who presented with sensory symptoms due to a pontine GBM.

## Case presentation

**Figure 1:**
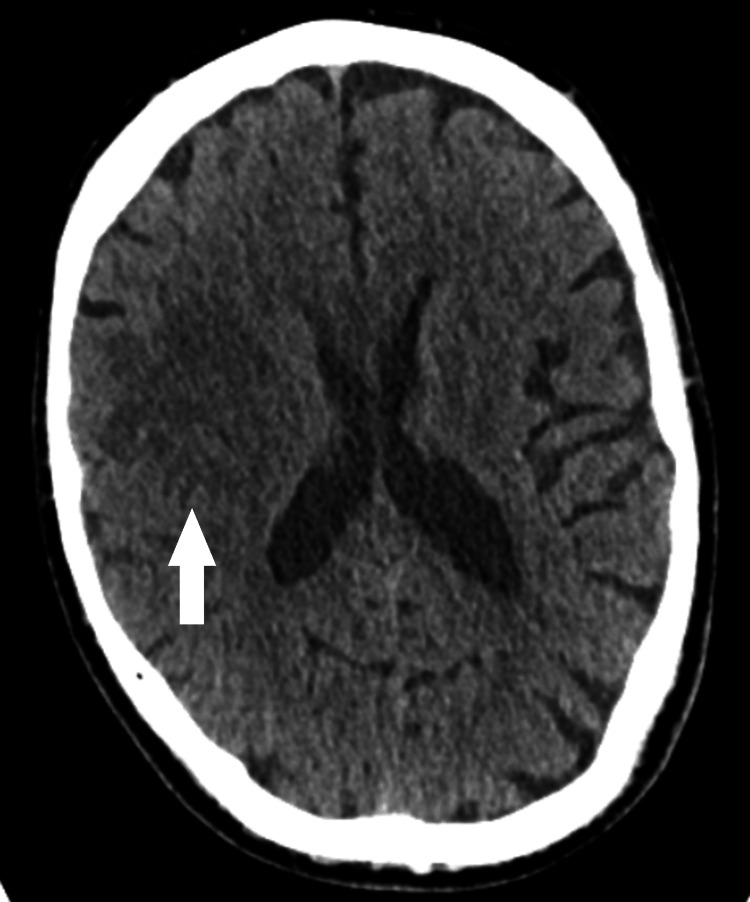
CT scan of the head A hypointensity in the right hemisphere was noted, which was not conclusive in terms of the underlying pathology CT: computed tomography

**Figure 2:**
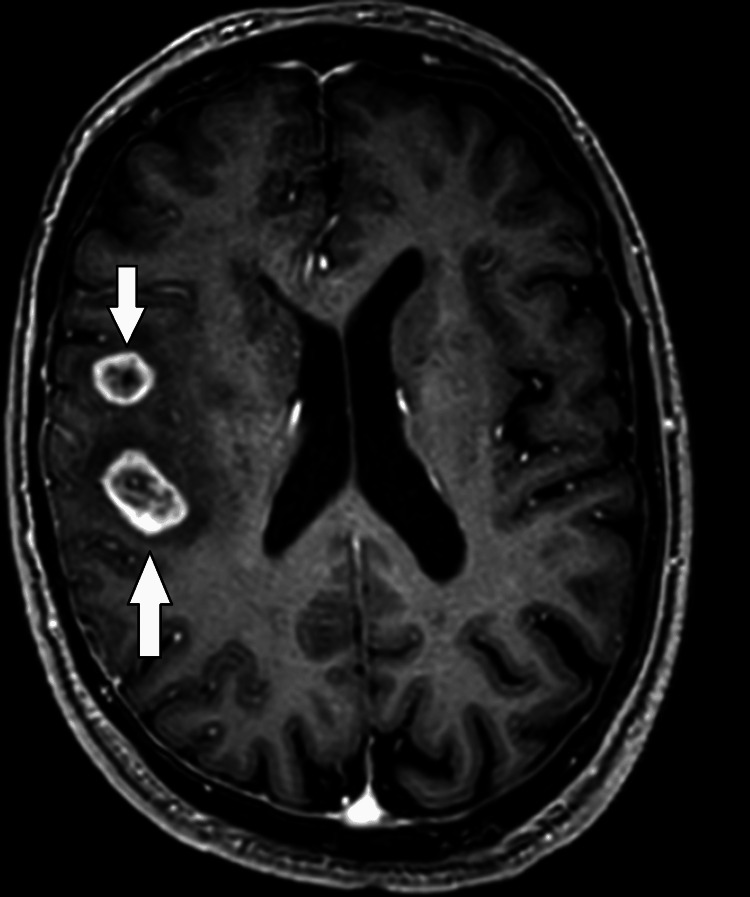
MRI of the head: T1-weighted sequence Peripheral discrete ring-enhancing lesions with central hypointensity were noted, raising suspicions of metastases MRI: magnetic resonance imaging

A 72-year-old man with a past medical history of atrial fibrillation (AF) presented with complaints of tingling and numbness in the left upper limb for one day. He was an independent and active individual who performed all his daily activities by himself. Given his age and history of AF, a noncontrast CT of the skull was done, which was inconclusive as shown in Figure 1. An MRI was also performed (Figure 2), which indicated a likely metastatic lesion, and hence further imaging to look for a primary tumour was advised. The patient was asymptomatic and was an in-patient only to undergo the scanning and to get a proper diagnosis.

A detailed history was taken from an oncology point of view but the patient denied any weight loss, fever, recent travel, family history, and other concerning symptoms apart from his initial symptoms. A contrast-enhanced CT scan of the thorax, abdomen, and pelvis was performed but did not reveal a primary tumour. Several physicians took an interest in the case and came up with various differentials, ranging from cerebral vasculitis to rare infections. However, all the blood tests including the complete blood count, liver function tests, kidney function tests, C-reactive protein, procalcitonin, and tumour markers, namely CA 19-9, alpha-fetoprotein, and prostate-specific antigen, were negative, and so was the patient's history.

**Figure 3:**
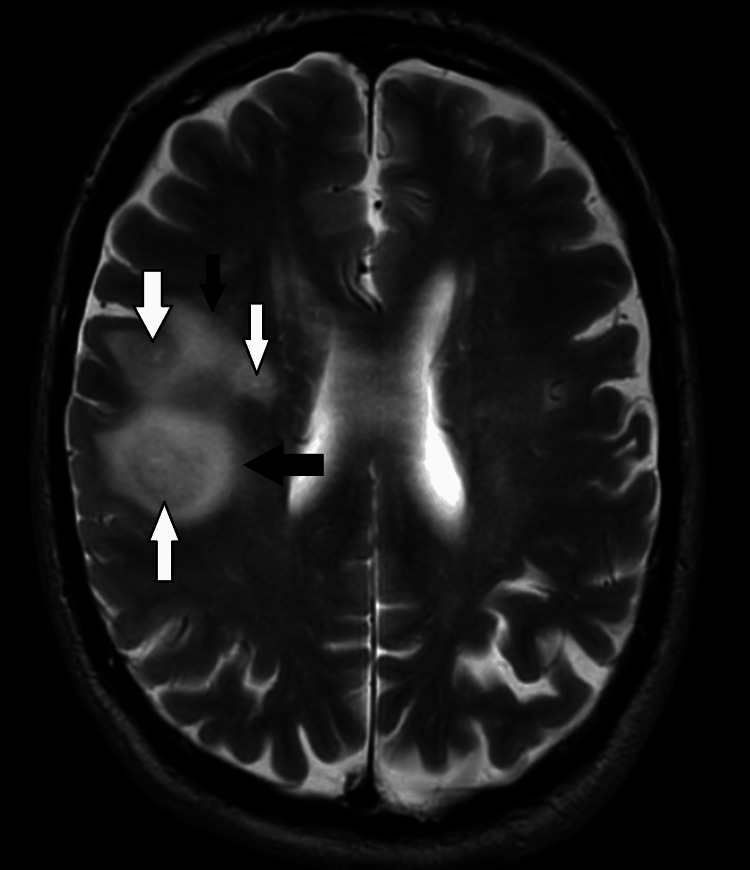
MRI of the head with contrast: T2-weighted sequence The white arrows show the necrotic core at the centre, while the black arrows show the perilesional edema - findings suggestive of GBM GBM: glioblastoma multiforme; MRI: magnetic resonance imaging

The scans were discussed at a neuroradiology MDT discussion, and an MRI with contrast was performed (Figure 3). After a thorough discussion, the patient was diagnosed with a grade IV glioblastoma - a primary tumour of the brain - which was confirmed histopathologically.

The patient was treated with two cycles of radiotherapy initially, followed by chemotherapy. Unfortunately, he died eight months after the start of the treatment due to a massive pulmonary embolism, followed by a nosocomial infection.

## Discussion

Atypical presentations of GBM are not uncommon. Cases with diagnostic difficulties have been described in the past, similar to our case. One such case was reported byZhang et al. in 2016 [9] where the patient was inappropriately treated for intracerebral infections and other illnesses based on the MRI findings, before eventually diagnosing GBM after a biopsy. That patient had a similar management plan to ours. Interesting papers by Sanli et al. [10] and Ekezie et al. [11] have described atypical presentations of glioblastoma, with the tumour presenting as sensory abnormalities in the former; Sanli et al.'s study discussed three different cases, one of which had left arm tingling and numbness and was treated for ulnar nerve entrapment, but later found to have GBM when the symptoms did not improve. The report by Ekezie et al. involved a 15-year-old male presenting with periumbilical tingling due to glioblastoma affecting sensory fibres due to its growth on the pons.

Glioblastoma is also feared due to its aggressive nature and quick progression. Due to this character of the tumour, by the time the symptoms appear, the mass may have already massively grown. One such case was published by Issac et al.[12]* *where the patient was initially thought to have a stroke, which on imaging was found to be a glioblastoma; it was treated with surgery initially, followed by another surgery in two weeks due to recurrence. The fate of the patient was not reported, but the article highlighted the violent nature of the tumour.

Given the varying presentation of the disease and the consequent diagnostic dilemma, further studies should be conducted about GBM to gain deeper insights into its clinical features and symptomatology. Through this report, we endeavour to shed light on this condition and aim to raise awareness among clinicians so that they can promptly diagnose and manage it.

## Conclusions

Glioblastoma is the most aggressive brain tumour and can present with a variety of symptoms. It is important to promptly diagnose this condition as it can progress very quickly. This is often challenging as the symptoms can be subtle and there are no specific risk factors associated with this tumour. It is also locally very invasive and commonly causes symptoms due to mass effect and compression of sensory or motor fibres, rather than compression of vital structures. The management comprises surgery as well as radiotherapy and chemotherapy, depending on the biopsy, immunohistochemistry, and cytogenetics of the tumour. Early diagnosis and treatment can significantly improve the prognosis in these patients.
